# Beyond Bacteria: The Impact of Protozoa on Periodontal Health

**DOI:** 10.3390/microorganisms13040846

**Published:** 2025-04-08

**Authors:** Bruno Pires Miranda, Marcos Tobias de Santana Miglionico, Rhagner Bonono dos Reis, Júlia de Castro Ascenção, Helena Lúcia Carneiro Santos

**Affiliations:** Laboratory of Parasitic Diseases, Oswaldo Cruz Institute, FIOCRUZ, Rio de Janeiro 21040-360, RJ, Brazil; brunopiresmiranda7@gmail.com (B.P.M.); miglioni@gmail.com (M.T.d.S.M.); bonono.rhagner@gmail.com (R.B.d.R.); julia_castro@id.uff.br (J.d.C.A.)

**Keywords:** *Entamoeba gingivalis*, *Acanthamoeba* spp., *Trichomonas tenax*, co-interactions

## Abstract

Until recently, bacterial species were the primary etiological factor of periodontal disease, but recent studies have shown that their effective removal from tooth surfaces does not necessarily prevent the progression of the disease. A logical hypothesis leads to the conclusion that various etiological factors contribute to the etiopathogenesis of this disease. Recent evidence also indicates protozoa as potential pathogens. In this review, the role of *Entamoeba gingivalis*, *Acanthamoeba* spp., and *Trichomonas tenax* in periodontal disease was analyzed, and the various aspects of the role of protozoa in the etiopathogenesis of periodontal disease, the bacterial–protozoa model of the disease, and the therapeutic implications were categorized. The multifactorial nature of periodontal disorders requires further research to better identify individuals at risk and prescribe earlier and more definitive treatments. The evidence supporting the role of protozoa in periodontal disease is compelling. These organisms are essential contributors to this complex disease. The key to developing more effective prevention and treatment strategies lies in understanding the intricate interactions between protozoa, bacteria, and the host. A better understanding of the protozoa aspect of periodontal disease progression could significantly change the future perspective of diagnosing, preventing, and treating periodontal disease.

## 1. Introduction

The human oral cavity possesses a dynamic microbial ecosystem, which is polymorphic and densely populated by bacteria, viruses, protozoa, and fungi associated with a diversity of ecological niches and colonization surfaces (gums, tongue, soft palate, buccal mucosa, and mineralized enamel surface teeth) [1]. Each of these habitats has its quirks that will influence the composition of the microbiota. Physiologically, the cavity possesses saliva, a complex fluid that removes excess bacteria and other microorganisms, diluting and homogenizing the members of the oral microbiota. Saliva contains approximately 109 micro-organisms per milliliter, continuously ingested through the diet. However, some bacteria organize and adhere to the buccal surface, forming the biofilm. The microbial community of a mature dental biofilm involves relatively solid intra- and interspecies and genera microbial interactions, which communicate by quorum sensing. Biofilms behave like an organized tissue, adapting quickly to environmental stress conditions. The microbiota balance is a determining factor for maintaining the individual’s health; added together, abiotic factors such as temperature, pH, redox potential, and nutrients beyond the immune system [2] that modulate colonization by certain microorganisms establish the formation of the resident microbiota.

Under healthy conditions, the oral microbiota has a harmonious relationship with the host and prevents the colonization of extrinsic bacteria that can even affect the systemic health of the host [3]. Smoking and eating foods containing various chemicals alter saliva’s osmotic and pH conditions, thus affecting the composition of the oral microbiome [1]. Because of this, certain microorganisms can move between commensalism and parasitism, which can trigger pathological outcomes such as dental caries and periodontal, pulp, and periapical diseases [4].

Therefore, knowing the oral microbiota is extremely important to understanding the mechanism of the formation of oral diseases. Periodontal disease refers to an inflammatory process in the supporting tissues of the teeth (free gingiva, attached gingiva, interdental papilla, alveolar mucosa, cementum, periodontal ligament, and alveolar bone). Microorganisms are responsible for reversible gingival inflammation (gingivitis) or permanently destroying tissues or parts of tissues, damaging connective tissue and alveolar bone. Several authors have reported *Porphyromonas gingivalis*, *Treponema denticola*, and *Tannerella forsythia* (the “red complex” in gingival plaques) as the primary agents involved in the pathogenesis of periodontitis [5,6]. For many years, studies have focused on the bacterial community of saliva. However, in recent decades, there has been a diffusion of knowledge about bacterial organization through the formation of biofilms, or bacterial plaques, in the oral cavity and on other surfaces.

Over the years, researchers have primarily studied the bacterial community present in our saliva. However, in recent decades, knowledge has increased about bacterial organization and how they form biofilms or bacterial plaques on various surfaces, including the oral cavity [3]. The biofilm structure comprises microbial communities immobilized in a matrix rich in extracellular substances, which protects against hostile environments and ensures access to nutrients necessary for survival. Bacterial biofilm is a multicellular structure playing a vital role in acquiring antibiotic resistance. Recently, analyses of periodontal biofilms in humans have revealed the presence of several microorganisms, including protozoa, *T. tenax*, and *E. gingivalis* [7,8,9]. *T. tenax* is associated with progressive states of periodontal disease [10,11,12].

The precise role of protozoa in periodontitis is yet to be determined, and there is a lack of information about it, which calls for further research. However, some studies have found a correlation between the increased occurrence of this protozoan and the progression of periodontal disease [13]. *P. gingivalis* and *Prevotella intermedia* are responsible for causing periodontal disease. They can survive and multiply inside *Acanthamoeba castellani*, present in the human oral cavity, nasal cavities, and throats. The cystic form of this amoeba is resistant to hospital antiseptics, which protects its endosymbionts from adverse environments. *Acanthamoeba* spp. can potentially exacerbate the virulence or pathogenic potential of microorganisms, leading to an infectious outcome for the host [14,15,16]. Thus, in this review, we analyze the role of *Entamoeba gingivalis*, *Acanthamoeba* spp., and *Trichomonas tenax* in periodontal disease and categorize the various aspects of the role of protozoa in its etiopathogenesis, in the bacterial–protozoal disease model, and in the therapeutic implications.

## 2. Materials and Methods

**Figure 1:**
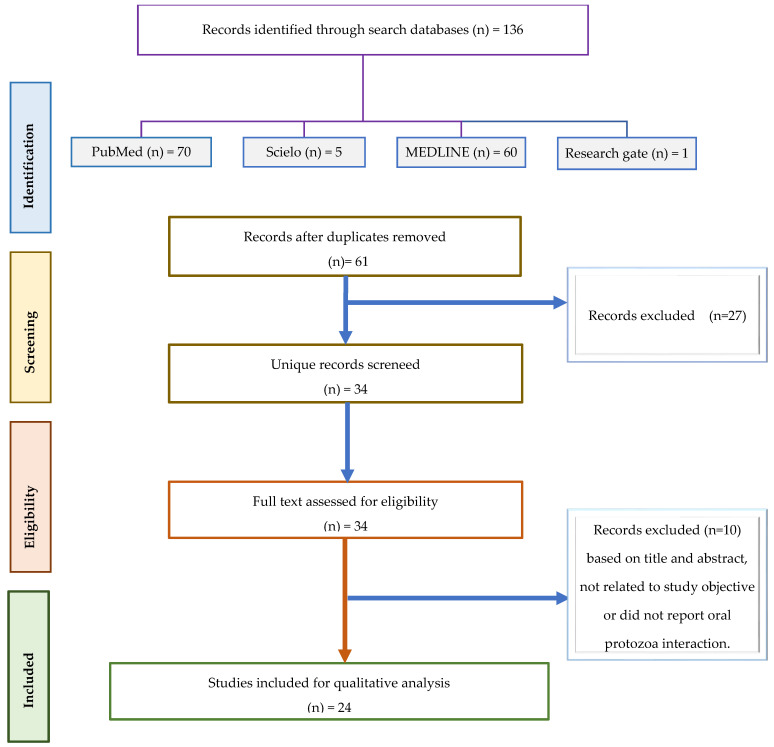
Flow diagram for selection of articles.

A thorough literature search was carried out through the online website International Databases, including Medline (via PubMed), SciELO, and Virtual Health Library (VHL), using the combination of keywords: (periodontal disease) AND (*Entamoeba gingivalis*); (periodontal disease) AND (*Acanthamoeba*); and (periodontal disease) AND (*Trichomonas tenax*). As this is a narrative review, no statistical analyses or measures to reduce bias through confounding analysis were applied. The search was narrowed down to English language studies without any date restrictions. In addition to this, Google Scholar was used to find gray literature, and the references of the included studies were manually checked to find more relevant studies. The studies selected for this analysis met the following inclusion criteria: involve the collection of dental biofilms (supra and/or subgingival), be in vitro studies with a detailed protocol, focus on the oral microbiome and its impacts on periodontitis, involve risk factors and approaches for detection of *Trichomonas tenax*, *Entamoeba gingivalis* and *Acanthamoeba* spp. Reviews and clinical studies were also considered. Any studies without abstracts, not in English, not relevant based on the title and abstract, or duplicates were excluded. Additionally, articles containing microorganisms other than bacteria and protozoa were not considered, and periodontal disease in animals was not included. A total of 136 papers were analyzed, and records were excluded if their titles and abstracts were not relevant to the study objectives or if they did not focus on oral-related topics (Figure 1).

## 3. Frequency of Protozoan Infection and Co-Interaction Protozoan-Bacteria

Recent findings suggest a multifaceted relationship between bacteria and protozoa in periodontal disease. The presence of protozoa may be linked to the dysbiotic state driven by pathogenic bacteria.

An analysis conducted by D’Ambrosio et al. [17] on the composition of the oral microbiome in users of fixed or removable dentures revealed that these prosthetic materials contribute to the development of oral biofilm. This is due to the decrease in salivary flow and pH, which alters the composition of the oral microbiota. The prosthesis covers a large area of the mouth, making it hard for saliva and the tongue’s cleaning action to reach, which can lead to the buildup of biofilm. In elderly people, oral tissue becomes less elastic and more fragile, making it more prone to injuries. This creates an environment where harmful bacteria and protists can thrive, leading to a shift from a healthy oral environment to an unhealthy one (“dysbiosis”) [18]. Diabetes is another factor that contributes to oral dysbiosis. Sucrose, the primary cariogenic sugar, can make the oral cavity more susceptible to colonization by microorganisms, increase plaque viscosity, and allow it to firmly attach to the teeth [19]. Fadhill et al. [20] discovered a higher percentage of *E. gingivalis* infection in diabetic individuals. This study also confirmed a statistically significant association between diabetes, hypertension, smoking, heart disease, and the prevalence of parasites causing periodontitis and gingivitis.

*E. gingivalis* can transition into opportunistic pathogens or free-living amoeba that become invasive in specific cases [19]. Studies in several countries have investigated the relationship between protozoa and periodontal disease. One of the studies in this scenario is that of Bonner et al. [21], in France, in which they studied the possibility of a link between the colonization of gingival crevices by *E. gingivalis* and this relationship with periodontal disease. Samples were collected from patients in eight dental clinics in France, resulting in 72 samples from periodontal pockets and 33 from healthy sites. The results demonstrated that *E. gingivalis* is significantly correlated with periodontal disease.

In Germany, Bao et al. [22], given the high frequency of *E. gingivalis* in inflamed periodontal disease sites, raised concerns about its supposed role in its pathogenesis. The researcher then investigated their infection strategies and virulence potential. The study revealed that, with the rupture of the epithelial barrier, *E. gingivalis* invaded the gingival tissue, where it moved and fed on the host cells. In primary gingival epithelial cells, infection with *E. gingivalis* (but not the oral bacterial pathogen *Porphyromonas gingivalis*) strongly increased the inflammatory cytokine IL8 (1900-fold) and the epithelial barrier gene MUC21.

The protozoan *E. gingivalis* has been found to inhibit cell proliferation and increase the expression of collagenase MMP13 in gingival fibroblasts. This suggests that *E. gingivalis* may play a significant role in the development of destructive forms of periodontal disease, which was previously underestimated. Studies have shown a high prevalence rate of *E. gingivalis* among patients with periodontal disease, indicating that it may be a risk factor for oral diseases. This prevalence was also observed in a study conducted in Turkey, which is the first report of detection and molecular subtyping of *E. gingivalis* in the country. Further research is needed to understand the specific role of this amoebae in the pathogenesis of periodontal disease [23,24].

In turn, another oral flagellated protozoan, *Trichomonas tenax*, has been associated with periodontal disease. In a review carried out by Marty et al. [18] in 2017 analyzing data from 47 studies, the prevalence of *T. tenax* was higher in patients with periodontal diseases than those with a healthy periodontium. This result showed a positive correlation with periodontal disease. Researchers suggest that this protozoan may be involved in the inflammatory process of gum disease. Some studies have provided initial evidence suggesting trichomonads’ potential role in the development of periodontal disease. However, further research is needed to fully understand their exact contribution. Moreover, trichomonads were found only in diseased sites with severe bone loss; in contrast, none were detected in healthy sites. Factors such as tooth mobility, heavy calculus, and severe attachment loss were linked to the presence of the protozoan. These results suggest an association between the presence of trichomonads and the severity of periodontal disease [25,26].

In accordance with this study, Bisson, Dridi, and Machouart’s [27] and Nor Azmi et al.’s [11] studies investigated the link between the *T. tenax* and periodontal disease. Their research found a strong correlation between the presence of this organism in diseased oral tissues and healthy ones. A higher prevalence of trichomonads was observed in diseased oral sites. Furthermore, the study revealed that protozoan can produce enzymes that break down periodontal tissues, suggesting a direct role in the development and progression of periodontal disease. Moreover, a study investigated the association between *T. tenax* and the severity of periodontal disease and diverse strains of *T. tenax,* with specific strains linked to more severe periodontal disease [25]. In turn, Bracamonte-Wolf et al.’s [28] study investigated the prevalence of this protozoan in patients with gingivitis and periodontal disease. *T. tenax* was identified in over half (56%) of the study participants, and the prevalence was significantly higher in patients with periodontal disease (70%) compared to those with gingivitis (35%). The presence of *T. tenax* increases with the severity of periodontal disease. Another interesting factor is that this protozoan has been shown to be associated with the presence of teeth, as it is absent in babies and completely edentulous patients [25,28].

Reassessing the coinfection of *E. gingivalis* and *T. tenax* in Periodontitis, Acurero Osorio et al. [29] determined the prevalence of both protozoa in the oral cavity of adult patients with and without periodontitis. *E. gingivalis* found in 10% of periodontitis patients but not in healthy controls, and *T. tenax* was found in 2% of periodontitis patients, associated with *E. gingivalis*. The authors pointed out the use of direct examination might have underestimated the prevalence of these protozoa, suggesting that the use of other techniques might lead to a higher rate of prevalence. Despite the limitations of the study, the findings suggest a possible association between *E. gingivalis* and periodontitis. This is supported by other studies that have reported a higher prevalence of *E. gingivalis* in patients with periodontal disease compared to healthy controls. Ghabanchi et al. [30] provided compelling evidence supporting a link between *E. gingivalis* and *T. tenax* and periodontal disease. Significantly higher prevalence of both parasites in patients with periodontal disease compared to healthy controls. This suggests a potential association between these protozoa and the development or progression of periodontal disease. Reinforcing the role of protozoa in oral health, these findings contribute to the growing body of evidence suggesting that protozoa, in addition to bacteria, play a role in periodontal disease.

Based on the studies in different regions, there is a strong correlation between the presence of *E. gingivalis* and *T. tenax* and periodontal disease. Studies show a significantly higher prevalence of these protozoa in individuals with periodontal disease compared to healthy controls. While some studies indicate a higher prevalence in gingivitis, others suggest a stronger association with periodontal disease. Moreover, prevalence rates can vary between different regions, suggesting potential influencing factors such as environmental conditions, lifestyle, and socio-economic status. However, although the exact mechanisms by which *E. gingivalis* and *T. tenax* contribute to periodontal disease are not fully understood, several hypotheses have been proposed: (i) these protozoa may directly damage periodontal tissues through their proteolytic enzymes and invasive properties; (ii) immune response modulation might induce an inflammatory response that contributes to tissue destruction; (iii) *E. gingivalis* may play a role in biofilm formation, creating a favorable environment for other pathogenic microorganisms; and (iv) the co-infection with both protozoa might enhance their pathogenic potential [31,32,33].

Some studies have presented highlight a significant discrepancy in the prevalence of *E. gingivalis* and *T. tenax* in relation to periodontal disease. Yaseen et al. [34] found a strong association between these protozoa, particularly *E. gingivalis*, and periodontal disease. The high prevalence rates in the study group compared to healthy controls support a potential causal link. On the other hand, Oladokun et al. [35] reported a very low prevalence of both protozoa in a Nigerian population with periodontal disease. Their conclusion challenges the established notion of these organisms as significant etiological factors in the disease. Different from the findings of Santos and Roldán [36] provide strong support for the involvement of *E. gingivalis* and *T. tenax* in the pathogenesis of periodontal disease. Their conclusion that these protozoa can interact with host cells, trigger inflammation, and contribute to tissue breakdown aligns with the emerging evidence in this field. These protozoa are not merely passive bystanders but active participants in the disease process or possess the ability to induce inflammation suggests a significant contribution to periodontal tissue destruction. And understanding the specific mechanisms by which these protozoa induce inflammation could lead to the development of new therapeutic strategies.

Another point of view to be evaluated is that there is a complex interplay between bacteria and protozoa in periodontal disease. Researchers investigated the prevalence and relative abundance of *E. gingivalis* in young patients with periodontal disease and its association with the subgingival microbial composition. In total, 120 sites from 60 patients were evaluated, and it was found that the prevalence and relative abundance of *E. gingivalis* increased significantly in sites with periodontal disease, in addition to being associated with *Porphyromonas* spp., *Treponema* spp., *Tannerella* spp., *Filifactor* spp., TG5, and *Desulfobulbus* spp. Thus, the authors concluded that *E. gingivalis* is closely associated with microbial dysbiosis, as well as with cytokines in the gingival crevicular fluid and can be considered an important pathogen in periodontal disease [37].

Another interesting study was carried out in France by Dubar et al. [12], who identified the presence of protozoa and bacteria, as well as associated clinical parameters, in patients with periodontal disease. The protozoa *Trichomonas tenax* and two subtypes of *Entamoeba gingivalis* (ST1 and an ST2 variant) and the following bacteria were identified as associated with the disease: *Porphyromonas gingivalis*, *Tannerella forsythia*, *Treponema denticola*, *Parvimonas micra*, *Fusobacterium nucleatum*, *Eubacterium nodatum*, and *Campylobacter rectus*. In addition, *T. tenax* alone was associated with *P. gingivalis*, *T. denticola*, and *E. nodatum*. The ST1 variant of *E. gingivalis* was significantly associated with gingival edema and a high total bacterial flora of subgingival biofilm samples. This is justified by the fact that amoebae need actively dividing bacteria to provide a favorable environment for their growth. However, no specific bacterial association was found with ST1. Therefore, the identification of the association of protozoa and bacteria in periodontal disease still requires further studies to better understand these interactions.

In turn, in their studies, Bonner et al. [21] and Garcia et al. [13] emphasized the importance of genetic variability within the *E. gingivalis* species. They found that the genetic distance between the ST1 and ST2 variants may indicate significant differences in their biology and pathogenicity. Meanwhile, Rayamajhee et al. [38] conducted a study characterizing the intracellular microorganisms of *Acanthamoeba* spp. in Australia and India. They used direct sequencing of amoeba 16S rRNA amplicons and discovered the potential for a sympatric lifestyle within *Acanthamoeba* spp. This highlights the crucial role of *Acanthamoeba* spp. as a host and transporter of potential human pathogens.

Some compelling evidence for a complex interplay between bacteria and protozoa in the pathogenesis of periodontal disease have been found. Periodontal pathogens like *P. gingivalis* and *Prevotella intermedia* can survive and replicate within free-living amoebas such as *A. castellanii*. This intracellular environment may protect bacteria from host defenses, allowing them to persist and potentially increase virulence. The coexistence of bacteria and protozoa, such as *E. gingivalis*-harboring periodontal pathogens, suggests a synergistic relationship in driving disease progression. A study revealed that *E. gingivalis* contains bacteria, including *P. gingivalis*, *Treponema denticola*, and *Tannerella forsythia* in patients with periodontal disease. Thus, this study indicates that the parasite–bacteria interaction can cause severe periodontal disease and alter the gingival microbiota [39]. These findings have significant implications for understanding and treating periodontal disease. Developing strategies to target both bacteria and protozoa could be essential for effective treatment, and assessing the role of protozoan in harboring and protecting pathogenic bacteria can provide insights into disease progression and severity.

## 4. Conclusions

Numerous studies from various countries consistently show a connection between the presence of *E. gingivalis* and *T. tenax* and the incidence of periodontal disease, indicating a significant correlation. These parasites have high virulence potential, as they invade gingival tissue, promote inflammation, inhibit cell proliferation, and regulate enzymes related to periodontal tissue degradation. Although there are some variations in study results from different regions, most studies emphasize the importance of further research on the relationship between these protozoa and periodontal diseases. To better understand the role of protozoa in periodontal disease, future research should focus on four main areas: (i) characterizing the inflammatory response to identify specific pro-inflammatory molecules induced by these protozoa; (ii) analyzing bacteria–protozoan interactions, particularly their interaction with immune cells such as neutrophils and macrophages; (iii) developing animal models to study how these protozoal infections influence the development of periodontitis; and (iv) conducting clinical trials to evaluate the effectiveness of antiprotozoal therapies. Genetic variations identified in *E. gingivalis* and the association of both protozoa with periodontopathogenic bacteria suggest a complex parasite–bacteria interaction that may influence the virulence and development of the disease. The results of analyzed studies show that *E. gingivalis*, in particular, not only acts as a potential pathogen in periodontal disease but also acts as a host for periodontopathogenic bacteria, aiding in their reproduction and increased virulence. Therefore, this symbiotic bacteria–protozoan relationship poses a risk for the emergence of periodontal disease. The evidence strongly supports the involvement of *E. gingivalis* and *T. tenax* in periodontal disease. A deeper understanding of their interactions with bacteria will be crucial for developing effective prevention and treatment strategies. Given the complex interplay between bacteria, protozoa, and the host immune response in periodontal disease, a multi-faceted approach to treatment is likely necessary.

## Data Availability

No new data were created or analyzed in this study.

## References

[1] Parras-Moltó M., López-Bueno A., Moya A., Brocal V.P. (2018). Methods for enrichment and sequencing of oral viral assemblages: Saliva, oral mucosa, and dental plaque viromes. The Human Virome: Methods and Protocols.

[2] Costalonga M., Herzberg M.C. (2014). The oral microbiome and the immunobiology of periodontal disease and caries. Immunol. Lett..

[3] Arweiler N.B., Netuschil L., Schwiertz A. (2016). The oral microbiota. Microbiota of the Human Body: Implications in Health and Disease.

[4] Siqueira J.F., Rôcas I.N., Seymour G.J., Cullinan M.P., Heng N.C.K. (2017). The oral microbiota in health and disease: An overview of molecular findings. Oral Biology: Molecular Techniques and Applications.

[5] Mohanty R., Asopa S.J., Joseph M.D., Singh B., Rajguru J.P., Saidath K., Sharma U. (2019). Red complex: Polymicrobial conglomerate in oral flora: A review. J. Fam. Med. Prim. Care.

[6] Socransky S.S., Haffajee A.D., Cugini M.A., Smith C., Kent R.L. (1998). Microbial com-plexes in subgingival plaque. J. Clin. Periodontol..

[7] Feki A., Molet B., Haag R., Kremer M. (1981). Les protozoaires de la cavité buccale humaine (corrélations épidémiologiques et pos-sibilités pathogéniques). J. Biol. Buccale.

[8] Kikuta N., Yamamoto A., Goto N. (1996). Detection and identification of *Entamoeba gingivalis* by specific amplification of rRNA gene. Can. J. Microbiol..

[9] Trim R.D., Skinner M.A., Farone M.B., Dubois J.D., Newsome A.L. (2011). Use of PCR to detect *Entamoeba gingivalis* in diseased gingival pockets and demonstrate its absence in healthy gingival sites. Parasitol. Res..

[10] Gharavi M.J., Hekmat S., Ebrahimi A., Jahani M.R. (2006). Buccal cavity protozoa in patients referred to the faculty of dentistry in Tehran, Iran. Iran. J. Parasitol..

[11] Nor Azmi N.J., Mohamad S., Shahidan W.N.S., Taib H., Mohamed Z., Osman E. (2024). Risk factors and approaches for detection of Trichomonas tenax, the silent culprit in periodontal disease: A narrative review. Saudi Dent. J..

[12] Dubar M., Zaffino M.L., Remen T., Thilly N., Cunat L., Machouart M.C., Bisson C. (2019). Protozoans in subgingival biofilm: Clinical and bacterial associated factors and impact of scaling and root planing treatment. J. Oral Microbiol..

[13] Garcia G., Ramos F., Maldonado J., Fernandez A., Yáñez J., Hernandez L., Gaytán P. (2018). Prevalence of two *Entamoeba gingivalis* ST1 and ST2-kamaktli subtypes in the human oral cavity under various conditions. Parasitol. Res..

[14] Janjalashvili T., Iverieli M. (2021). Frequency of presence of periodontopathogenic bacteria in the periodontal pockets. Georgian Med. News..

[15] Akya A., Pointon A., Thomas C.J. (2009). Interactions between *Acanthamoeba castellanii* and bacterial pathogens: A review. Parasitology.

[16] Wang Y., Jiang L., Zhao Y., Ju X., Wang L., Jin L., Fine R.D., Li M. (2023). Biological characteristics and pathogenicity of *Acanthamoeba*. Front. Microbiol..

[17] D’Ambrosio F., Santella B., Di Palo M.P., Giordano F., Lo Giudice R. (2023). Characterization of the Oral Microbiome in Wearers of Fixed and Removable Implant or Non-Implant-Supported Prostheses in Healthy and Pathological Oral Conditions: A Narrative Review. Microorganisms.

[18] Marty M., Lemaitre M., Kémoun P., Morrier J.-J., Monsarrat P. (2017). *Trichomonas tenax* and periodontal diseases: A concise review. Parasitology.

[19] Ibrahim S., Abbas R.S. (2012). Evaluation of *Entamoeba gingivalis* and *Trichomonas tenax* in patients with periodontitis and gingivitis and its correlation with some risk factors. J. Baghdad Coll. Dent..

[20] Fadhil Ali Malaa S., Abd Ali Abd Aun Jwad B., Khalis Al-Masoudi H. (2022). Assessment of *Entamoeba gingivalis* and *Trichomonas tenax* in Patients with Chronic Diseases and its Correlation with Some Risk Factors. Arch. Razi Inst..

[21] Bonner M., Amard V., Bar-Pinatel C., Charpentier F., Chatard J.M., Desmuyck Y., Ihler S., Rochet J.P., de La Tribouille V.R., Saladin L. (2014). Detection of the amoeba *Entamoeba gingi-valis* in periodontal pockets. Parasite.

[22] Bao X., Wiehe R., Dommisch H., Schaefer A.S. (2020). Entamoeba gingivalis Causes Oral Inflammation and Tissue Destruction. J. Dent. Res..

[23] Badri M., Olfatifar M., Abdoli A., Houshmand E., Zarabadipour M., Abadi P.A., Johkool M.G., Ghorbani A., Eslahi A.V. (2021). Current Global Status and the Epidemiology of *Entamoeba gingivalis* in Humans: A Systematic Review and Meta-analysis. Acta Parasitol..

[24] Örsten S., Şahin C., Yılmaz E., Akyön Y. (2023). First molecular detection of *Entamoeba gingivalis* subtypes in individuals from Turkey. Pathog. Dis..

[25] Benabdelkader S., Andreani J., Gillet A., Terrer E., Pignoly M., Chaudet H., Aboudharam G., La Scola B. (2019). Specific clones of *Trichomonas tenax* are associated with periodontitis. PLoS ONE.

[26] Bisson C., Lec P.H., Blique M., Thilly N., Machouart M. (2018). Presence of trichomonads in subgingival biofilm of patients with per-iodontitis: Preliminary results. Parasitol. Res..

[27] Bisson C., Dridi S.M., Machouart M. (2019). Assessment of the role of *Trichomonas tenax* in the etiopathogenesis of human periodontitis: A systematic review. PLoS ONE.

[28] Bracamonte-Wolf C., Orrego P.R., Muñoz C., Herrera D., Bravo J., Gonzalez J., Varela H., Catalán A., Araya J.E. (2019). Observational cross-sectional study of *Trichomonas tenax* in patients with periodontal disease attending a Chilean university dental clinic. BMC Oral Health.

[29] Acurero Osorio E.M., Maldonado Ibáñez A.B., Ibáñez C.M., Bracho Mora A.M., Parra J., Urdaneta Y., Urdaneta M. (2009). *Entamoeba gingivalis* y *Trichomonas tenax* en cavidad bucal de pacientes de la Clínica Integral del Adulto de la Facultad de Odontología, Maracai-bo, Venezuela. Rev. Soc. Venez. Microbiol..

[30] Ghabanchi J., Zibaei M., Afkar M.D., Sarbazie A.H. (2010). Prevalence of oral *Entamoeba gingivalis* and *Trichomonas tenax* in patients with periodontal disease and healthy population in Shiraz, southern Iran. Indian J. Dent. Res..

[31] Derikvand N., Mahmoudvand H., Sepahvand A., Baharvand P., Kiafar M.M., Chiniforush N., Ghasemi S.S. (2018). Frequency and associated risk factors of *Entamoeba gingivalis* and *Trichomonas tenax* among patients with periodontitis in Western Iran. J. Res. Med. Dent. Sci..

[32] Yazar S., Çetinkaya Ü., Hamamcı B., Alkan A., Şişman Y., Esen Ç., Kolay M. (2016). Investigation of *Entamoeba gingivalis* and *Trichomonas tenax* in Periodontitis or Gingivitis Patients in Kayseri. Turk. Parazitol. Derg..

[33] Araújo-Rosa J.A., dos Santos-Fernandez M., Soares-Vieira I., Riscala-Madi R., Moura de Melo C., Costa da Cunha-Oliveira C. (2020). Detection of Oral *Entamoeba gingivalis* and *Trichomonas tenax* in Adult Quilombola Population with Periodontal Disease. Odovtos.

[34] Yaseen A., Mahafzah A., Dababseh D., Taim D., Hamdan A.A., Al-Fraihat E., Hassona Y., Şahin G.Ö., Santi-Rocca J., Sallam M. (2021). Oral Colonization by *Entamoeba gingivalis* and *Trichomonas tenax*: A PCR-Based Study in Health, Gingivitis, and Periodontitis. Front. Cell. Infect. Microbiol..

[35] Oladokun A.O., Ogboru P., Opeodu O.I., Lawal A.O., Falade M.O. (2023). Prevalence of *Entamoeba gingivalis* and *Trichomonas tenax* among patients with periodontal disease attending Dental Clinic, University College Hospital, Ibadan. Trop. Parasitol..

[36] Santos J.O., Roldán W.H. (2023). *Entamoeba gingivalis* and *Trichomonas tenax*: Protozoa parasites living in the mouth. Arch. Oral Biol..

[37] Jiao J., Bie M., Xu X., Duan D., Li Y., Wu Y., Zhao L. (2022). *Entamoeba gingivalis* is associated with periodontal conditions in Chinese young patients: A cross-sectional study. Front. Cell. Infect. Microbiol..

[38] Rayamajhee B., Willcox M., Sharma S., Mooney R., Petsoglou C., Badenoch P.R., Sherchan S., Henriquez F.L., Carnt N. (2024). Zooming in on the intracellular microbiome composition of bacterivorous Acanthamoeba isolates. ISME Commun..

[39] Huang J.M., Ting C.C., Chen Y.C., Yuan K., Lin W.C. (2021). The first study to detect co-infection of *Entamoeba gingivalis* and perio-dontitis-associated bacteria in dental patients in Taiwan. J. Microbiol. Immunol. Infect..

